# Neutrophil to lymphocyte ratio influences impact of steroids on efficacy of immune checkpoint inhibitors in lung cancer brain metastases

**DOI:** 10.1038/s41598-021-85328-w

**Published:** 2021-04-05

**Authors:** Adam Lauko, Bicky Thapa, Mayur Sharma, Baha’eddin Muhsen, Addison Barnett, Yasmeen Rauf, Hamid Borghei-Razavi, Vineeth Tatineni, Pradnya Patil, Alireza Mohammadi, Samuel Chao, Erin S. Murphy, Lilyana Angelov, John Suh, Gene H. Barnett, Amy S. Nowacki, Nathan Pennell, Manmeet S. Ahluwalia

**Affiliations:** 1 Cleveland Clinic Lerner College of Medicine At Case Western Reserve University, 9500 Euclid Ave, CA-51, Cleveland, OH 44195 USA; 2 Foedtert and Medical College of Wisconsin, Milwaukee, WI USA; 3 Department of Neurological Surgery, University of Louisville, Louisville, KY USA; 4 Rosa Ella Burkhart Brain Tumor and Neuro-Oncology Center, Taussig Cancer Institute, Cleveland Clinic, Cleveland, OH USA; 5 Department of Neurological Surgery, Neurological Institute, Cleveland Clinic, Cleveland, OH USA; 6 Department of Neurological Surgery, Cleveland Clinic Florida, Weston, FL USA; 7 Department of Medicine, Summa Health, Akron, OH USA; 8 Department of Quantitative Health Sciences, Lerner Research Institute, Cleveland Clinic, Cleveland, OH USA; 9 Department of Medical Oncology, Taussig Cancer Institute, Cleveland Clinic, Cleveland, OH USA; 10 Department of Radiation Oncology, Taussig Cancer Institute, Cleveland Clinic, Cleveland, OH USA; 11 Department of Medical Oncology, Miami Cancer Institute, Baptist Health South Florida, Miami, FL USA

**Keywords:** Non-small-cell lung cancer, CNS cancer

## Abstract

Steroids are often utilized to manage patients with non-small cell lung cancer brain metastases (NSCLCBM). Steroids and elevated neutrophil-to-lymphocyte ratio (NLR) have been associated with decreased overall survival (OS) in patients treated with immune checkpoint inhibitors (ICI). We retrospectively investigated patients treated with ICI after the diagnosis of NSCLCBM at a single tertiary care institution examing the impact of steroids and NLR. Overall survival (OS) and intracranial progression-free survival (PFS) were analyzed. 171 patients treated with ICI for NSCLCBM were included. Thirty-six received steroids within 30 days of the start of ICI, and 53 patients had an NLR ≥ 5 before the start of ICI. Upfront steroids was associated with decreased OS on multivariable analysis (median OS 10.5 vs. 17.9 months, *p* = .03) and intracranial PFS (5.0 vs. 8.7 months, *p* = .045). NLR ≥ 5 was indicative of worse OS (10.5 vs. 18.4 months, *p* = .04) but not intracranial PFS (7.2 vs. 7.7 months, *p* = .61). When NLR and upfront steroids are modeled together, there is a strong interaction (*p* = .0008) indicating that the impact of steroids depended on the patient’s NLR. In a subgroup analysis, only in patients with NLR < 4 was there a significant difference in OS with upfront steroids (26.1 vs. 15.6 months, *p* = .032). The impact of steroids on the efficacy of ICI in patients with NSCLCBM is dependent on the patient's NLR underscoring its importance in these patients. Patients with a low NLR, steroid use decreases the efficacy of ICI. These results can inform clinicians about the impact of steroids in patients treated with ICI.

## Introduction

Approximately 10–30 percent of patients with Non-Small Cell Lung Cancer (NSCLC) will develop brain metastases (BM) during the course of their disease^1,2^. Historically, systemic chemotherapies have shown minimal intracranial efficacy^3–5^. However, the development of targeted and immune-modulating therapies have changed treatment paradigms in NSCLCBM. In patients without targetable driver mutations, immune checkpoint inhibitors (ICIs) are commonly-used systemic therapeutic options for managing intracranial metastases^6,7^. While early trials provided some evidence for intracranial efficacy^8–10^, the wide-scale use of ICI in patients with NSCLC has provided an opportunity for retrospective investigation^11^.

As a result of the widespread use of ICI in patients with NSCLCBM, there is a growing interest in identifying factors predictive of therapeutic response in this patient population. Steroids are frequently used for symptomatic management of patients with NSCLCBM to treat fatigue, dyspnea, decreased appetite, and other cancer-related symptoms, however, their immune-supressive mechanism of action is hypothesized to antagonize ICI activity. The first evidence of this was reported in a phase 2 clinical trial in melanoma BM, which demonstrated a better response to ICI in patients who did not receive upfront steroids and were neurologically asymptomatic when compared to patients who received steroids^12^. Since then, studies have shown that the use of steroids within the first 30 days of initiating ICI is associated with a decrease in OS^13,14^. However, others have argued that this association exists only because the patients with a worse prognosis are more likely to receive steroids^15^. There is a paucity of literature showing direct correlation between steroid use and outcome in patients receiving ICI for NSCLCBM.

Another clinical marker, neutrophil to lymphocyte ratio (NLR), has been proposed as a surrogate for immune activity^16–20^. Multiple studies in NSCLC have reported an elevated NLR at the start of immunotherapy is associated with a poor prognosis^21–24^. However, there is limited data investigating the impact of NLR in patients with BM and its interaction with steroid use.

In this study, we investigated the impact of upfront steroids and NLR on OS and PFS in a cohort of patients who began ICI after the diagnosis of NSCLCBM, a patient cohort with a universaly poor prognosis. We hypothesized that even within this group, upfront steroid use and elevated NLR would result in a decrease in OS and intracranial PFS.

## Methods

### Patient selection and treatment

This IRB-approved retrospective cohort study included consecutive NSCLCBM patients treated with ICI with the diagnosis of BM from 2015 to 2019 at a single tertiary-care institution. Patient information was gathered retrospectively from charts and therefore the IRB waived the informed consent requirement. Patient characteristics, diagnostic information, and treatment details were abstracted from the shared electronic medical record. This included Karnofsky Performance Score (KPS), number of BMs, presence of extra-cranial metastases, neurologic symptoms, age, PD-L1 status in the primary tumor, and molecular markers (EGFR, ALK, PDL1, KRAS). Treatment details included the number of cycles of ICI and local intracranial treatment such as whole brain radioation therapy (WBRT), surgery, and stereotactic radiosurgery (SRS) at any time after diagnosis of BM.

All patients who were > 18 years of age with pathologically confirmed diagnosis of NSCLC and treated with one or more of the ICIs (nivolumab, pembrolizumab, atezolizumab, or ipilimumab) were included in our study. Patients were followed in the clinic with surveillance MRIs every 2–3 months. Intracranial progression was defined utilizing a modified iRANO brain metastases criteria^25,26^. Progression was defined as a greater than 20% increase in the sum of the longest diameter of target lesions, the unequivocal progression of enhancing non-target lesions, new lesions, or death. For cases of potential pseudoprogression, the judgement of the hospitals interdisciplinary tumor board was utilized. All patients who received ICI before their diagnosis of BM were excluded from our study.

### Steroid and NLR data

Upfront steroid use was defined as any outpatient prescription of steroids within the first 30 days of the beginning of immunotherapy. NLR was calculated from the complete blood count (CBC) on the day of the start of ICI or the most recent CBC with differential data within one month prior to the start of ICI.

### Statistical analysis

For baseline characteristics, continuous data were compared across cohorts with Student’s t-test or Wilcoxon rank-sum test, while categorical data were compared with Chi-squared tests. The Kaplan–Meier method was used to estimate OS and PFS from the start of ICI. Patients were grouped according to upfront steroid usage and NLR, with time-to-event differences tested according to the Wilcoxon test as we desired to give more weight to early events. Multivariable analysis for OS and PFS was conducted utilizing Cox-proportional hazards models. Models were adjusted for the following covariates: age at diagnosis of BM, presence of extracranial metastases, number of BM, KPS, and the presence of neurological symptoms at diagnosis of BM. Covariates were chosen based upon previously described risk factors for local failure and variables prognostic for survival. Multicollinearity was assessed by examining the variance inflation factors. In addition to the main effects model, NLR was allowed to modify the effect of steroid usage with the inclusion of a cross-product term.

Test for covariate interaction between NLR and steroid use was performed. NLR was treated as both a continuous variable in some analysis and as a dichotomous variable with a cut-off between 4–6 in others, as reported in previous studies^14,24^. In order to determine the individual contributions of both the neutrophil and lymphocyte counts, patients were grouped according to a neutrophil count cut-off of 8000 (cells/mm^3^) or lymphocyte count of 1000 (cell/mm^3^) and compared with respect to OS and PFS. Similar comparisons were conducted after keeping both variables continuous^27^.

Statistical significance was defined as a *p*-value of less than 0.05. JMP Pro version 14 statistical software (SAS Institute, Cary, NC) was utilized for analysis. GraphPad Prism 8 (San Diego, California) was utilized for the generation of images.

### Ethics approval

This retrospective chart review study involving human participants was in accordance with the ethical standards of the institutional and national research committee and with the 1964 Helsinki Declaration and its later amendments or comparable ethical standards. The Human Investigation Committee (IRB) of the Cleveland Clinic approved this study (Protocol #09-911).

## Results

### Patient characteristics

Within the study period, 171 patients were treated with ICI after the diagnosis of NSCLCBM. The ICI received included nivolumab (n = 101), pembrolizumab (n = 52), atezolizumab (n = 14), durvalumab (n = 3) and ipilimumab (n = 1). Within our cohort, the grade prognostic assessment (GPA) remains a strong predictor of OS as patients with a GPA of 2 or greater had a better prognosis than patients with a GPA of less than 2 (median survival 21 vs 13.4 months, *p* = 0.03). Thirty-six patients were treated with upfront steroids with a median duration of 2 months (range 0.1–12) and a median prednisone equivalent dose of 27 mg per day (range 5–107), with 29 of these patients receiving dexamethasone. Patient demographics and covariates that are known to be prognostic of OS in NSCLCBM are documented in Table 1. There was no significant difference in these variables between the upfront steroids group and the control group. More patients who received upfront steroids had neurologic symptoms at the diagnosis of BM, but this was only marginally significant (68.6% vs. 51.9%, *p* = 0.08). After splitting the cohort by NLR status, we observed that patients with an NLR ≥ 5 were more likely to be male patients (*p* = 0.006). Additionally, patients with NLR < 5 were more likely to have undergone surgical resection of an intracranial lesion at any time after diagnosis of BM (*p* = 0.003). On univariate OS analysis of previously correlated variables, none of the variables showed statistical significance (Supplemental Table S1).

**Table 1:** Patient characteristics. (A) Patients separated into cohorts based upon upfront steroid usage within 30 days of start of immunotherapy. (B) Patients separated into cohorts based upon NLR status (≥ 5 vs. < 5). *p*-value: a. Student’s t-test, b. Pearson Chi-square test c. Wilcoxon sum test.

(A)	No upfront steroids (n = 135)	Upfront steroids (n = 36)	*p* value
Age, mean (SD)	64 (9.5)	62 (9.2)	.17^a^
% Male	44%	44%	.94^b^
% Caucasian	86%	81%	.42^b^
%PD-L1 Positive	79.6	92.3	.25^b^
% with Extracranial Metastases	50%	53%	.80^b^
Baseline Number of Intracranial Lesions, mean (SD)	2.8 (3.4)	2.4 (1.6)	.39^a^
KPS (median (Q_1_–Q_3_))	90 (80–90)	85 (70–90)	.28^c^
**% with Neurological Symptoms at Diagnosis**	**52%**	**69%**	**.08**^**b**^
Mean Number of SRS treatments (SD)	1.85 (1.31)	1.91 (1.20)	.80^a^
% that received WBRT	24%	28%	.61^b^
% Craniotomy	15%	17%	.61^b^
% EGFR mutation	9%	10%	.91^b^
% ALK rearrangement	2%	3%	.56^b^
% KRAS mutation	49%	55%	.62^b^

(B)	NLR ≥ 5 (n = 53)	NLR < 5 (n = 95)	*p* value
Age, mean (SD)	65 (9.1)	63 (9.6)	.21^a^
**% Male**	**60%**	**37%**	**.006**^**b**^
% Caucasian	83%	86%	.72^b^
%PD-L1 Positive	77.3	82.9	.59^b^
% with Extracranial metastases	57%	47%	.19^b^
Baseline Number of Intracranial Lesions, mean (SD)	3.3 (4.2)	2.4 (2.4)	.14^a^
KPS (median (Q_1_–Q_3_))	90 (80–90)	90 (80–90)	.47^c^
% with Neurological Symptoms at Diagnosis	51%	56%	.53^b^
Mean Number of SRS treatments (SD)	**1.5 (1.0)**	**2.0 (1.3)**	**.01**^**a**^
% that received WBRT	28%	22%	.40^b^
**% Craniotomy**	**2%**	**20%**	**.003**^**b**^
% EGFR mutation	14%	8%	.32^b^
% ALK rearrangement	2%	0%	.16^b^
% KRAS mutation	57%	43%	.22^b^
% Upfront Steroids	23%	17%	.39^b^

### Impact of upfront steroids

**Figure 1:**
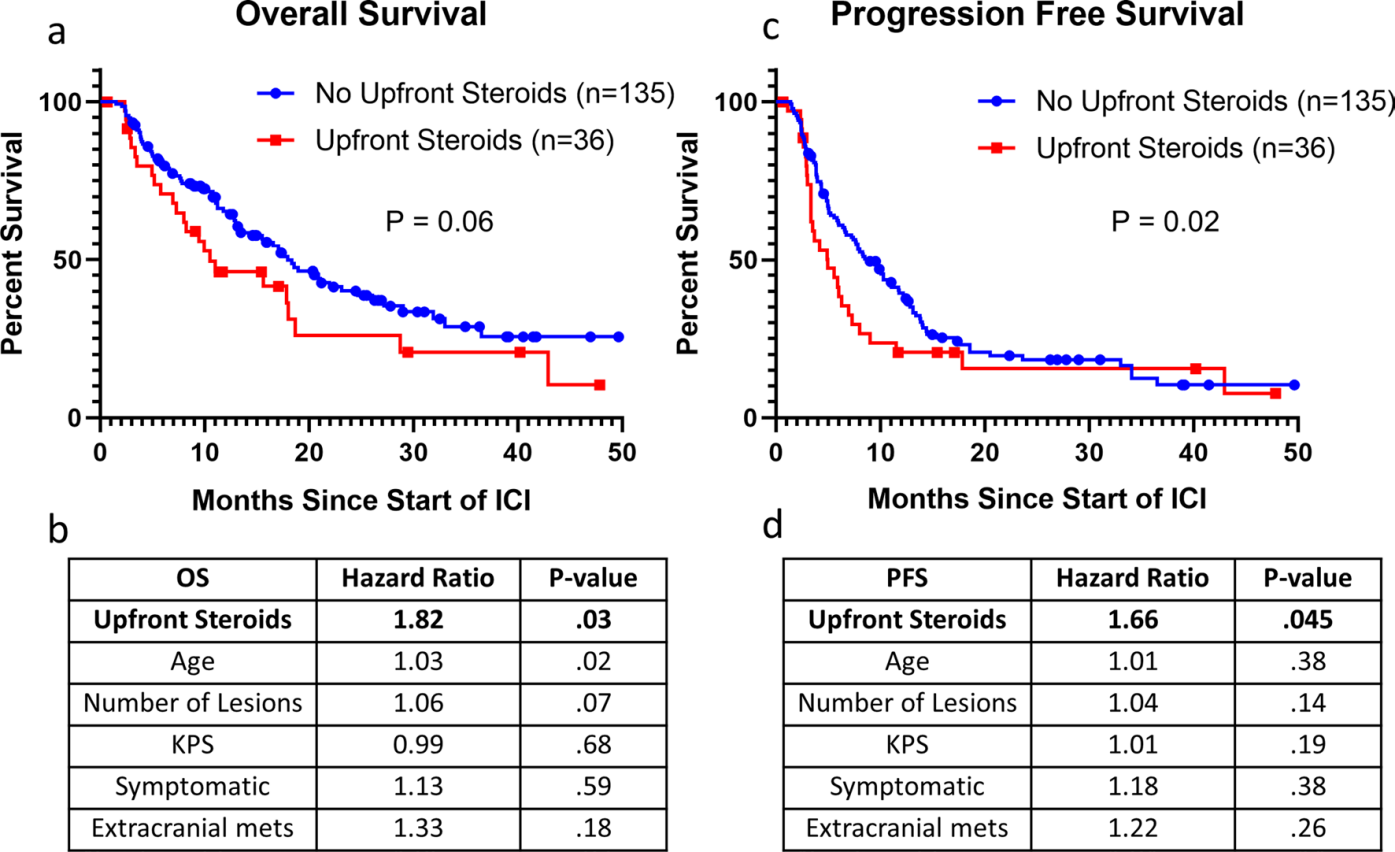
Upfront steroids decrease the efficacy of immune checkpoint inhibitors in NSCLCBM. (**a**) Overall Survival differences between those treated with or without upfront steroids. (**b**) Multivariable analysis of overall survival. (**c**) Progression-free survival differences between those treated with or without upfront steroids. (**d**) Multivariable analysis of progression-free survival. (**a**) and (**c**) group differences tested with the Wilcoxon test. (**b**) and (**d**) estimates from Cox proportional hazards models and the associations tested with effect likelihood ratio tests.

The use of upfront steroids was marginally associated with a lower median OS (10.5 months) compared to patients who did not receive upfront steroids (17.9 months) (unadjusted *p* = 0.06) (Fig. 1a). After adjustment, the use of upfront steroids (HR: 1.82, *p* = 0.03) and increasing age (HR: 1.03, *p* = 0.02) were significantly associated with reduced OS (Fig. 1b). In patients that received upfront steroids, the dose of steroids did not correlate with OS (HR = 0.99, *p* = 0.57). Additionally, we separated patients by the duration of received steroids into greater than or equal to 2 months compared to less than 2 months and found no association (*p* = 0.23). The same held for dichotomization at 1 and 3 months (*p* = 0.69 and *p* = 0.46 respectively).

In terms of intracranial PFS, the use of upfront steroids led to a decrease in median PFS from 8.7 months to 5 months (*p* = 0.02) (Fig. 1c). After adjustment, the use of upfront steroids was significantly associated with decreased intracranial PFS (HR:1.66, *p* = 0.045) (Fig. 1d).

### Impact of NLR

**Figure 2:**
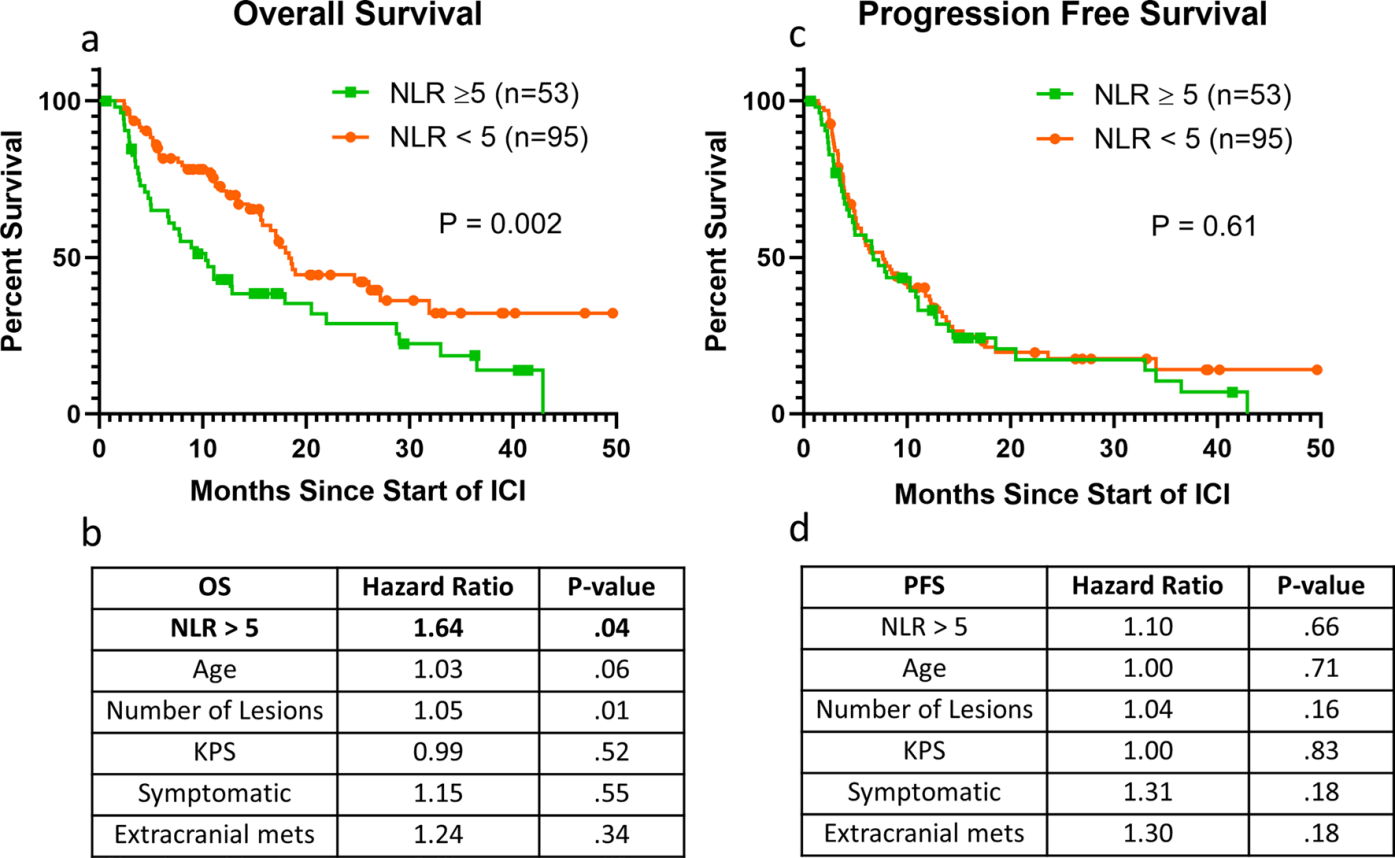
NLR is associated with overall survival after start of ICI but not associated with PFS. (**a**) Overall Survival differences between those with NLR ≥ 5 versus < 5. (**b**) Multivariable analysis of overall survival. (**c**) Progression-free survival differences between those with NLR ≥ 5 versus < 5. (**d**) Multivariable analysis of progression-free survival. (**a**) and (**c**) group differences tested with the Wilcoxon test. (**b**) and (**d**) estimates from Cox proportional hazards models and the associations tested with effect likelihood ratio tests.

NLR data was available for 148 patients in our cohort (87%). NLR of ≥ 5 was indicative of worse OS (Fig. 2a). The median OS with an NLR of ≥ 5 was 10.5 months versus 18.4 months with an NLR < 5 (unadjusted *p* = 0.002). After adjustment, NLR of ≥ 5 was significantly associated with decreased OS (HR:1.64, *p* = 0.04) (Fig. 2b). However, unlike upfront steroids, NLR was not predictive of intracranial PFS (*p* = 0.61) (Fig. 2c). After adjustment, there remained no evidence of an association among NLR and PFS. (Fig. 2d).

Due to the arbitrary nature of the NLR cut-off of 5, a cut-off of 4 and 6 were also explored. The NLR cut-off of 4 demonstrated a significant association with OS (*p* < 0.001) and a non-significant association with PFS (*p* = 0.58). The NLR cut-off of 6 was also associated with significant OS (*p* < 0.0001) and a non-significant association with PFS (*p* = 0.17). When NLR was used as a continuous variable, NLR did not show a significant association with OS (HR = 1.03, *p* = 0.07), suggesting there may be a range of values where NLR does not impact OS after start of ICI (nonlinear association).

To determine the individual contribution of the neutrophil count, a cut-off of 8000 cell/mm^3^ was utilized. Patients with a neutrophil count over 8000 cell/mm^3^ had a significantly worse OS compared to those less than 8000 cell/mm^3^ with a median survival of 7.2 months versus 18.7 months (*p* = 0.0001). Neutrophil count used as a continuous variable also led to a HR of 1.065 (95% CI: 1.02–1.10, *p* = 0.002). When investigating the impact of lymphocyte count utilizing a cut-off of 1000 cell/mm^3^, we found no evidence of a difference in OS (*p* = 0.40) or PFS (*p* = 0.13). Additionally, when lymphocyte count was used as a continuous variable, the HR for OS was 0.91 (95% CI: 0.65–1.16, *p* = 0.52).

### Impact of upfront steroids is dependent on NLR

**Figure 3:**
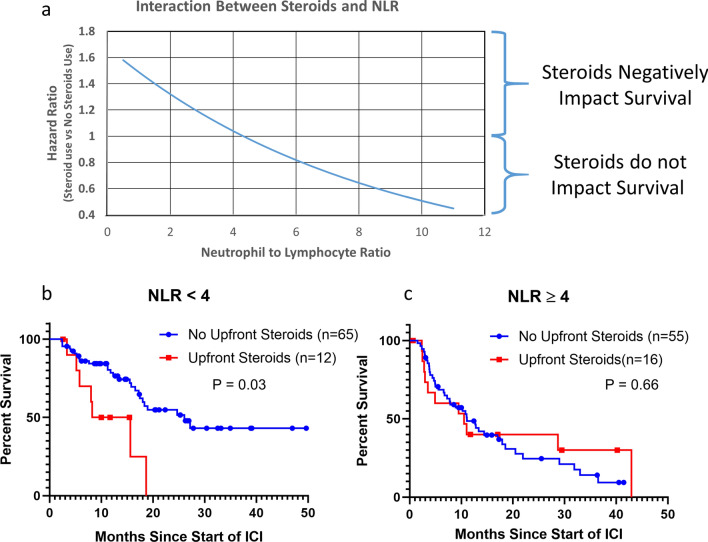
Association of upfront steroids with OS is dependent on NLR. (**a**) A model was generated utilizing known predictors of OS in NSCLCBM, upfront steroid use, NLR as well as an interaction term between NLR and upfront steroids. The hazard ratio of upfront steroid use was calculated at varying NLRs. Impact of upfront steroids was then analyzed based on NLR less than 4 (**b**) and NLR greater than or equal to 4 (**c**).

In order to determine if the impact of steroids was dependent of NLR we created a multivariable model that included an interaction term between upfront steroid use and continuous NLR and found a strong significant interaction (*p* < 0.001) (Table 2). Utilizing this model, the HR for upfront steroid usage was calculated across NLR values ranging from 0.5 to 11 and graphed in Fig. 3a. These results demonstrate that steroids have a large negative impact on survival in patients with a low NLR. However, steroids have a minimal and possibly beneficial effect on survival in patients with a high NLR.

**Table 2:** Multivariable analysis of OS including upfront steroids, NLR and an interaction term between upfront steroids and NLR. Estimates from Cox proportional hazards models and the associations tested with effect likelihood ratio tests.

	Hazard ratio	*p* value
Age	1.03	.05
Number of Baseline Intracranial Lesions	1.05	.14
Neurologic Symptoms	1.01	.96
KPS	1.00	.79
Extra-cranial Metastases	1.29	.26
Steroid	–	.08
NLR	–	.40
Interaction term (Steroid X NLR)	–	**.0008**

We then separated our cohort into two groups based on an NLR of above or below 4, based on the value in Fig. 3a where the hazard ratio crosses 1. In patients with an NLR below 4, steroid use was significantly associated with decreased OS (26.1 vs. 15.6 months, *p* = 0.032) (Fig. 3b). This was confirmed on multivariable analysis (*p* = 0.02) (Supplemental Table S2a). Whereas, in patients with an NLR ≥ 4, steroids had no impact on OS (11.1 vs. 10.5 months, *p* = 0.66) (Fig. 3c), and this held on multivariable analysis (HR = 0.79, *p* = 0.59) (Supplemental Table S2b).

### Local therapy

The interaction between radiation therapy and ICI has been previously documented^11^. In our cohort, 126 patients of 171 received only SRS, eight patient received only WBRT, 34 patients received WBRT and SRS and zero patients only underwent a craniotomy after the diagnosis of BM. Only six patients had a craniotomy after starting ICI. Impact of SRS versus no SRS was investigated but no significant findings were observed. Of interest, 17 patients received WBRT after of starting ICI and these patients had a far worse prognosis. When WBRT after start of ICI was added our models for overall survival, there was a significant hazard ratio ranging from 2.25–2.44. This did not alter any of the earlier conclusions (Supplemental Table S3).

## Discussion

ICIs are the standard of care in NSCLC and have rapidly improved the outcomes of these patients. Since the start of ICI clinical trials, determining which patients will respond best to ICI has been a goal of clinicians and scientists. For example, reseachers have found that patients negative for the expression of the two most frequent HLA-A alleles a worse prognosis in NSCLC treated with ICI^28,29^. Others have found that commonly utilized medications like systemic antibiotics and proton-pump inhibitors can decrease the efficacy of ICIs^30^.

### Upfront steroids

Our study aimed to investigate the impact of steroids and NLR in NSCLCBM patients. In concordance with the literature, our study showed that the use of upfront steroids was associated with reduced OS and PFS in NSCLCBM treated with ICI^11–14^. Arbour et al.^13^ reported that upfront use of steroids (> 10 mg of prednisone) was associated with decreased OS in patients with NSCLC. However, Ricciuti et al.^15^ showed that the negative impact of steroids was only seen in patients who received steroids for cancer-related palliation, suggesting that the observed difference may be driven by the poor prognosis of the steroid subgroup and not diminished efficacy of ICI^15^. Our study furthers this work by investigating the impact of steroids in a group of NSCLCBM patients who are likely to have a poor prognosis. Using this methodology, we found that steroids do impact the intracranial progression of disease and OS.

### Neutrophil to lymphocyte ratio

Our results demonstrated a strong association between OS and NLR across multiple cut-offs, as previously reported in patients with NSCLC treated with ICI^21–24^. However, association between OS and NLR in patients with NSCLC is independent of ICI^31^. A recent meta-analysis (n = 27 articles) reported a correlation between OS and NLR in NSCLC patients treated with chemotherapy, bevacizumab, immunotherapy, and targeted therapies^32–35^. Additionally NLR levels correlate with survival in NSCLCBM patients treated with SRS^36^, or undergoing surgery^37^. Our study adds to the literature by demonstrating that while NLR is a strong predictor of OS, there is little evidence that NLR predicts the intracranial response to ICI. Intracranial PFS is a measure of therapeutic response and contrary to our hypothesis, the absence of difference in PFS suggests NLR does not directly impact the efficacy of ICI. Based on our intracranial finding and the fact that NLR correlates with OS across treatments in NSCLC, we propose that NLR should be understood as a prognostic factor, much like graded prognostic assessment, and not a measure specific to immune activation.

### Interaction between steroids and neutrophil to lymphocyte ratio

We investigated interaction between baseline NLR and upfront steroid use to determine whether NLR could predict the impact of steroids on ICI efficacy. Our data suggests that there is a range of NLR values, below 4 where steroid use impacts the efficacy of ICI, likely through the suppression of the immune response. However, as NLR increases, steroids no longer diminish the efficacy of ICI. This may be attributed to the fact that the prognosis in these patients is so poor that ICI is likely minimally effective. Our data suggest that in healthier patients (lower NLR), upfront steroids appear to significantly diminish the efficacy of ICI.

### Local therapy

The relationship between ICI and radiotherapy has been demonstrated to be important^11^. We found that patients receiving WBRT with ICI had a poor prognosis. We hypothesize that this is highlighting a population of patients receiving palliative WBRT compared to those receiving SRS but the study was not designed to investigate this further. Additionally, we investigated the impact of SRS our outcome and found no significant results, which is likely a function of only 11 patients not receiving SRS.

### Strengths and limitations

The retrospective nature of this project comes with its inherent limitations, such as selection, treatment, and elimination bias. Within our study, patients were not randomized, NLR was not available for all patients and the collection of KPS and neurologic symptoms relied on documentation in the electronic medical record. The patients received five different ICI therapies. We investigated the importance of type of ICI and saw no correlation with or impact on any of our result, however, there remains a possibility each drug behaves differently. Finally, NLR is a crude marker of inflammation and more complex models of inflammatory status have been developed that integrate a wide range of cytokines and may provide a clearer picture. Nevertheless, our study provides insight into the interaction between steroids, NLR, and clinical outcomes in a large cohort of patients at a tertiary care multidisciplinary NeuroOncology center.

## Conclusion

NLR is associated with OS in patients with NSCLCBM treated with ICI, but demonstrated no association with PFS. In patients with low NLR, upfront steroid treatment decreases the efficacy of ICI. Our data suggests that when clinicians are balancing improving a patients quality of life with steroids and their fear of diminishing the efficacy of ICI, NLR is an objective and widely available clinical marker that can be utilized to determine in which patients steroids may be detrimental to OS. This research needs further prospective validation in a larger randomized controlled study.

## Supplementary Information

Supplementary Information.

## Abbreviations

NSCLCBM: Non-small cell lung cancer brain metastases
ICI: Immune checkpoint inhibitors
BM: Brain metastases
NLR: Neutrophil to lymphocyte ratio
WBRT: Whole-brain radiation therapy
SRS: Stereotactic radiosurgery
OS: Overall survival
PFS: Progression-free survival
KPS: Karnofsky performance status
PD-L1: Programmed death ligand-1
EGFR: Epidermal growth factor receptor
ALK: Anaplastic lymphoma receptor tyrosine kinase
KRAS: V-Ki-Ras2 Kirsten Rat Sarcoma 2 Viral Oncogene Homolog
