# Author Correction: Neutrophil to lymphocyte ratio influences impact of steroids on efficacy of immune checkpoint inhibitors in lung cancer brain metastases

**DOI:** 10.1038/s41598-021-96915-2

**Published:** 2021-09-08

**Authors:** Adam Lauko, Bicky Thapa, Mayur Sharma, Baha’eddin A. Muhsen, Addison Barnett, Yasmeen Rauf, Hamid Borghei‑Razavi, Vineeth Tatineni, Pradnya Patil, Alireza Mohammadi, Samuel Chao, Erin S. Murphy, Lilyana Angelov, John Suh, Gene H. Barnett, Amy S. Nowacki, Nathan Pennell, Manmeet S. Ahluwalia

**Affiliations:** 1 Cleveland Clinic Lerner College of Medicine At Case Western Reserve University, 9500 Euclid Ave, CA‑51, Cleveland, OH 44195 USA; 2 Foedtert and Medical College of Wisconsin, Milwaukee, WI USA; 3 Department of Neurological Surgery, University of Louisville, Louisville, KY USA; 4 Rosa Ella Burkhart Brain Tumor and Neuro‑Oncology Center, Taussig Cancer Institute, Cleveland Clinic, Cleveland, OH USA; 5 Department of Neurological Surgery, Neurological Institute, Cleveland Clinic, Cleveland, OH USA; 6 Department of Neurological Surgery, Cleveland Clinic Florida, Weston, FL USA; 7 Department of Medicine, Summa Health, Akron, OH USA; 8 Department of Quantitative Health Sciences, Lerner Research Institute, Cleveland Clinic, Cleveland, OH USA; 9 Department of Medical Oncology, Taussig Cancer Institute, Cleveland Clinic, Cleveland, OH USA; 10 Department of Radiation Oncology, Taussig Cancer Institute, Cleveland Clinic, Cleveland, OH USA; 11 Department of Medical Oncology, Miami Cancer Institute, Baptist Health South Florida, Miami, FL USA

Correction to: *Scientific Reports* https://doi.org/10.1038/s41598-021-85328-w, published online 05 April 2021

The original version of this Article contained an error in the spelling of the author Baha’eddin A. Muhsen, which was incorrectly given as Baha’eddin Muhsen.

As a result, in the Author Contribution section,

“The experimental design was done by A.L., B.T., M.S., P.P., Y.R., V.T., A.M., S.C., E.S.M., L.A., J.S., G.H.B., A.S.N., N.P., M.S.A. Data collection was performed by A.L., B.T., A.B., B.M., H.B.R.”

now reads:

“The experimental design was done by A.L., B.T., M.S., P.P., Y.R., V.T., A.M., S.C., E.S.M., L.A., J.S., G.H.B., A.S.N., N.P., M.S.A. Data collection was performed by A.L., B.T., A.B., B.A.M., H.B.R.”

The original Article and accompanying Supplementary Information file have been corrected.

