# Author Correction: Neutrophil to lymphocyte ratio influences impact of steroids on efficacy of immune checkpoint inhibitors in lung cancer brain metastases

**DOI:** 10.1038/s41598-022-05488-1

**Published:** 2022-01-18

**Authors:** Adam Lauko, Bicky Thapa, Mayur Sharma, Baha’eddin A. Muhsen, Addison Barnett, Yasmeen Rauf, Hamid Borghei-Razavi, Vineeth Tatineni, Pradnya Patil, Alireza Mohammadi, Samuel Chao, Erin S. Murphy, Lilyana Angelov, John Suh, Gene H. Barnett, Amy S. Nowacki, Nathan Pennell, Manmeet S. Ahluwalia

**Affiliations:** 1 Cleveland Clinic Lerner College of Medicine At Case Western Reserve University, 9500 Euclid Ave, CA-51, Cleveland, OH 44195 USA; 2 Foedtert and Medical College of Wisconsin, Milwaukee, WI USA; 3 Department of Neurological Surgery, University of Louisville, Louisville, KY USA; 4 Rosa Ella Burkhart Brain Tumor and Neuro-Oncology Center, Taussig Cancer Institute, Cleveland Clinic, Cleveland, OH USA; 5 Department of Neurological Surgery, Neurological Institute, Cleveland Clinic, Cleveland, OH USA; 6 Department of Neurological Surgery, Cleveland Clinic Florida, Weston, FL USA; 7 Department of Medicine, Summa Health, Akron, OH USA; 8 Department of Quantitative Health Sciences, Lerner Research Institute, Cleveland Clinic, Cleveland, OH USA; 9 Department of Medical Oncology, Taussig Cancer Institute, Cleveland Clinic, Cleveland, OH USA; 10 Department of Radiation Oncology, Taussig Cancer Institute, Cleveland Clinic, Cleveland, OH USA; 11 Department of Medical Oncology, Miami Cancer Institute, Baptist Health South Florida, Miami, FL USA

Correction to: *Scientific Reports* 10.1038/s41598-021-85328-w, published online 05 April 2021

The original version of this Article contained an error in the Funding section.

“No funding for this manuscript.”

now reads:

“This work was partially funded by the Ruth L. Kirschstein National Research Service Award (NRSA) Individual Fellowship F30CA250254 (AL).”

The original Article has been corrected.

