# Supplementary material for: Neutrophil to lymphocyte ratio influences impact of steroids on efficacy of immune checkpoint inhibitors in lung cancer brain metastases
Source: Sci Rep. 2021 Apr 5;11:7490. doi: 10.1038/s41598-021-85328-w (PMC8021556; doi:10.1038/s41598-021-85328-w)
Supplement: Supplementary file 1 — Supplementary Information. [file 41598_2021_85328_MOESM1_ESM.docx]

**Neutrophil to Lymphocyte Ratio Influences Impact of Steroids on Efficacy of Immune Checkpoint Inhibitors in Lung Cancer Brain Metastases**

Adam Lauko BS^1^, Bicky Thapa MD^2^, Mayur Sharma MD^3^, Baha’eddin Muhsen MD^4,5^, Addison Barnett MS^4^, Yasmeen Rauf MD^4^, Hamid Borghei-Razavi MD PhD^6^, Vineeth Tatineni MD^7^ Pradnya Patil MD^9^, Alireza Mohammadi MD^1,4,5^, Samuel Chao MD^4,10^, Erin S Murphy MD^4,10^, Lilyana Angelov MD^1,4,5^, John Suh MD^4,10^, Gene H Barnett MD ^1,4,5^, Amy S Nowacki PhD^1,8^, Nathan Pennell MD PhD^9^, Manmeet S. Ahluwalia MD, MBA*^1,4,9^

1. Cleveland Clinic Lerner College of Medicine at Case Western Reserve University, Cleveland, OH
2. Foedtert and Medical College of Wisconsin, Milwaukee, WI
3. Department of Neurological Surgery, University of Louisville, Louisville, KY
4. Rosa Ella Burkhart Brain tumor and Neuro-Oncology Center, Taussig Cancer Institute, Cleveland Clinic, Cleveland, OH,
5. Department of Neurological Surgery, Neurological Institute, Cleveland Clinic, Cleveland, OH
6. Department of Neurological Surgery, Cleveland Clinic Florida, Weston FL
7. Department of Medicine, Summa Health, Akron, OH
8. Department of Quantitative Health Sciences, Lerner Research Institute, Cleveland Clinic, Cleveland, OH
9. Department of Medical Oncology, Taussig Cancer Institute, Cleveland Clinic, Cleveland, OH
10. Department of Radiation Oncology, Taussig Cancer Institute, Cleveland Clinic, Cleveland, OH

*Coresponding Author:

**Manmeet Ahluwalia**

Cleveland Clinic

9500 Euclid Ave, CA-51

Cleveland, OH 44195

Phone: (216) 280 2412

[ahluwam@ccf.org](mailto:ahluwam@ccf.org)

**Supplemental Tables**

| **Variable** | **P-value** |
| --- | --- |
| Age | .06 |
| Number of Baseline Intracranial Lesions | .19 |
| Neurologic Symptoms | .61 |
| KPS | .38 |
| Extra-cranial Metastases | .19 |

**Table S1:** Univariate associations of variables previously identified in the literature to predict overall survival (OS) in patients with NSCLCBM.

| **Table S2A NLR < 4** | **HR** | **P-value** |
| --- | --- | --- |
| **Upfront Steroid** | **3.1** | **.02** |
| Age | 1.03 | .12 |
| Number of Baseline Intracranial Lesions | 1.04 | .59 |
| Neurologic Symptoms | 1.16 | .71 |
| KPS | 0.99 | .74 |
| Extra-cranial Metastases | 1.11 | .78 |

| **Table S2B NLR ≥ 4** | **HR** | **P-value** |
| --- | --- | --- |
| **Upfront Steroid** | **0.79** | **.59** |
| Age | 1.02 | .40 |
| Number of Baseline Intracranial Lesions | 1.05 | .10 |
| Neurologic Symptoms | 1.30 | .43 |
| KPS | 0.99 | .70 |
| Extra-cranial Metastases | 1.43 | .24 |

**Table S2:** OS multivariable analysis of baseline NLR below 4 (A) and baseline NLR greater than or equal to 4 (B). Estimates from Cox proportional hazards models and the associations tested with effect likelihood ratio tests.

| **Table S3A Upfront Steroids** | **HR** | **P-value** |
| --- | --- | --- |
| **Upfront Steroids** | **1.81** | **.03** |
| **WBRT** | **2.33** | **.006** |
| **Age** | **1.03** | **.01** |
| Number of Baseline Intracranial Lesions | 1.06 | .09 |
| KPS | 1 | .67 |
| Symptomatic | .95 | .8 |
| Extra-cranial Metastases | 1.32 | .18 |

| **Table S3B NLR** | **HR** | **P-value** |
| --- | --- | --- |
| **NLR > 5** | **1.66** | **.03** |
| **WBRT** | **2.25** | **.016** |
| **Age** | **1.03** | **.048** |
| Number of Baseline Intracranial Lesions | 1.05 | .132 |
| KPS | .99 | .55 |
| Symptomatic | 1.20 | .44 |
| Extra-cranial Metastases | 1.21 | .40 |

| **Table S3C Interaction Term** | **HR** | **P-value** |
| --- | --- | --- |
| **Interaction Term (Steroid X NLR)** | **-** | **.0005** |
| **WBRT** | **2.44** | **.01** |
| NLR | - | .34 |
| Upfront Steroids | - | .08 |
| Age | 1.02 | .04 |
| Number of Baseline Intracranial Lesions | 1.05 | .15 |
| KPS | 1 | .81 |
| Symptomatic | 1.06 | .8 |
| Extra-cranial Metastases | 1.28 | .28 |

**Table S3:** OS multivariable analysis including WBRT with ICI of upfront Steroids (A), baseline NLR greater than or equal to 5 (B), and an interaction term between upfront steroids and NLR (C). Estimates from Cox proportional hazards models and the associations tested with effect likelihood ratio tests.
